# Rho Kinase Inhibition with Fasudil in the SOD1^G93A^ Mouse Model of Amyotrophic Lateral Sclerosis—Symptomatic Treatment Potential after Disease Onset

**DOI:** 10.3389/fphar.2017.00017

**Published:** 2017-01-31

**Authors:** René Günther, Alexander Balck, Jan C. Koch, Tobias Nientiedt, Michael Sereda, Mathias Bähr, Paul Lingor, Lars Tönges

**Affiliations:** ^1^Department of Neurology, University Medicine GöttingenGöttingen, Germany; ^2^Department of Neurology, Technische Universität DresdenDresden, Germany; ^3^Institute of Neurogenetics, University of LübeckLübeck, Germany; ^4^Department of Neurogenetics, Max-Planck-Institute of Experimental MedicineGöttingen, Germany; ^5^Department of Clinical NeurophysiologyGöttingen, Germany; ^6^Cluster of Excellence Nanoscale Microscopy and Molecular Physiology of the BrainGöttingen, Germany; ^7^Department of Neurology, St. Josef-Hospital, Ruhr University BochumBochum, Germany

**Keywords:** ROCK, Fasudil, SOD1, mouse model of ALS, neuroprotection

## Abstract

Despite an improved understanding of the genetic background and the pathomechanisms of amyotrophic lateral sclerosis (ALS) no novel disease-modifying therapies have been successfully implemented in clinical routine. Riluzole still remains the only clinically approved substance in human ALS treatment with limited efficacy. We have previously identified pharmacological rho kinase (ROCK) inhibitors as orally applicable substances in SOD1.G93A transgenic ALS mice (SOD1^G93A^), which are able to extend survival time and improve motor function after presymptomatic treatment. Here, we have evaluated the therapeutic effect of the orally administered ROCK inhibitor Fasudil starting at a symptomatic disease stage, more realistically reflecting the clinical situation. Oral Fasudil treatment was initiated at a symptomatic stage at 80 days of life (d80) with 30 or 100 mg/kg body weight in both female and male mice. While baseline neurological scoring and survival were not influenced, Fasudil significantly improved motor behavior in male mice. Spinal cord pathology of motoneurons (MN) and infiltrating microglial cells (MG) at disease end-stage were not significantly modified. Although treatment after symptom onset was less potent than treatment in asymptomatic animals, our study shows the therapeutic benefits of this well-tolerated substance, which is already in clinical use for other indications.

## Introduction

Amyotrophic lateral sclerosis (ALS) is a devastating neuromuscular disease, which is characterized by the degeneration of the first and second motoneuron (MN) resulting in severe muscle weakness. Within a few years after initial diagnosis death is often caused by global respiratory insufficiency. The neurodegenerative process in ALS comprises the loss of spinal MNs and their axons as well as the destruction of neuromuscular junctions, where distinct pathological changes can be observed first. Some authors thus consider ALS as a distal axonopathy (Moloney et al., 2014). The mechanisms of ALS pathogenesis are multifactorial and include protein aggregation, oxidative stress, excitotoxicity, mitochondrial dysfunction, disturbances in RNA metabolism, and impaired axonal transport (Robberecht and Philips, 2013; Peters et al., 2015). Moreover, microglia (MG), astroglia, and oligodendroglia may determine the onset and the dynamics of disease progression (Philips and Robberecht, 2011; Brites and Vaz, 2014).

Recently, the spectrum of human familial ALS forms has been broadened by the discovery of several novel gene mutations (Vucic et al., 2014). While genetic mutations in the human superoxide dismutase 1 (SOD1) were the first to be identified (Rosen et al., 1993), further pathogenic mutations have also been found in Fused in sarcoma (FUS), TAR DNA-binding protein 43 (TDP-43), or in the gene C9orf72 (Turner et al., 2013). Some of these familial ALS gene mutations have been employed to establish new transgenic mouse models of ALS. Because of its close recapitulation of the progression of human disease, however, the SOD1^G93A^ mouse model of ALS has evolved as a standard model for the evaluation of therapeutic effects in preclinical studies (Turner and Talbot, 2008). Several neuroprotective agents have been found to delay the onset of clinical disease and to prolong the disease course in ALS mice, but only the glutamate antagonist Riluzole was successfully transferred into clinical practice (Benatar, 2007). Unfortunately, Riluzole has only limited effects in human ALS patients prolonging life only by a few months (Stewart et al., 2001). Therefore, the need for more potent disease-modifying therapies of ALS remains.

Rho kinases (ROCK) are serine/threonine kinases that are known to modulate cytoskeletal structure through phosphorylation of LIM kinases, myosin light chain or the ezrin/radixin/moesin protein complex (Tönges et al., 2011). Fasudil, 1-(5-Isoquinolinylsulfonyl) homopiperazine, belongs to the isoquinoline series of ROCK inhibitors and contains as structural feature an isoquinoline and homopiperazine ring, which are connected by a sulphonyl group. The inhibition of ROCK is of competitive type and takes place at the ATP binding site (Jacobs et al., 2006). Fasudil is one of the most thoroughly evaluated ROCK inhibitors and has been studied in various models of neurodegenerative disease (Bowerman et al., 2012; Tönges et al., 2012, 2014; Li et al., 2013; Takata et al., 2013; Zhao Y. F. et al., 2015; Tatenhorst et al., 2016). In these, a good oral bioavailability and effective penetration of the blood brain barrier were shown (Hattori et al., 2004; Takata et al., 2013; Tönges et al., 2014). More importantly, our group and others have recently demonstrated that inhibition of ROCK with pharmacological small molecule inhibitors strongly fosters the regenerative response in the lesioned central nervous system (CNS; Lingor et al., 2008; Planchamp et al., 2008; Bermel et al., 2009) and can activate intracellular neuroprotective and anti-apoptotic signaling cascades such as the Akt pathway (Tönges et al., 2012; Takata et al., 2013; Zhao Y. et al., 2015). In SOD1^G93A^ ALS mice ROCK activity was shown to be increased and phosphorylated Akt levels were decreased (Takata et al., 2013; Golko-Perez et al., 2016). Treatment with the ROCK inhibitor Fasudil strengthened Akt signaling and led to increased survival of motoneurons if treatment was started presymptomatically (Takata et al., 2013). Furthermore, we could recently demonstrate in studies in SOD1^G93A^ ALS mice that the presymptomatic use of the small-molecule ROCK inhibitor Fasudil is able to extend survival time and improve motor function (Tönges et al., 2014). Protein levels of ROCK2 as well as of its downstream targets LIMK1 and cofilin2 have been shown to be significantly increased in skeletal muscle biopsies from ALS patients compared to age-matched controls (Conti et al., 2014). Interestingly, phosphorylated Akt levels were found to be decreased in muscle biopsies of ALS patients in comparison to their control counterparts (Léger et al., 2006).

Here, we have now conducted an oral treatment study with the ROCK inhibitor Fasudil in symptomatic SOD1^G93A^ mice, because in clinical reality treatment initiation before symptom onset will hardly be feasible for sporadic ALS cases. As starting point for treatment initiation we have chosen day of life 80 (d80) based on first signs of disease in neurological examination and in electrophysiological measurements. Fasudil was applied in two different dosages in both male and female animals. The clinical disease course and performance in behavioral tests was closely monitored and was compared to the immunohistological analysis of the spinal cord MN survival and MG infiltration at end-stage.

## Materials and methods

### Animal housing, breeding, genotyping, and application of fasudil

All animal experiments were carried out according to the regulations of the local animal research council (Niedersächsisches Landesamt für Verbraucherschutz und Lebensmittelsicherheit, Oldenburg, Germany) and legislation of the State of Lower Saxony, Germany. High-copy number B6/SJL-Tg(SOD1 G93A)1Gur/J (Gurney et al., 1994) were obtained from Jackson Labs (Stock Number 002726; Bar Harbor, USA). The colony was maintained by crossing B6/SJL males harboring the transgene with wild-type B6/SJL females. Housing of animals was performed under a 12 h light/12 h dark cycle with free access to food and water. For genotyping, tail biopsies of 21-day-old mice were subjected to a standardized PCR protocol (Jackson Labs). Probe sequences were: hSOD1-forward, CATCAGCCCTAATCCATCTGA; hSOD1-reverse, CGCGACTAACAATCAAAGTGA; both SOD1^G93A^ female and male mice were used in the experiments. Fasudil (“F-4660,” LC Labs, Woburn, Massachusetts, USA) was orally administered at a concentration of 30 mg/kg body weight per day (subsequently termed as Fas30) or at a concentration of 100 mg/kg body weight per day (Fas100) in drinking water. Control groups received drinking water without supplementation (Veh).

### Electrophysiological analysis

Nerve conduction velocities (NCV) and compound muscle action potentials (CMAP) were determined as previously described (Zielasek et al., 1996). Time points of analysis were 70 and 100 days of age. Briefly, mice were anesthetized using an intraperitoneal injection of ketamine (100 mg/kg body weight) and xylazine (8 mg/kg body weight). CMAP and distal motor latencies (DML) were recorded from the intrinsic foot muscles using steel electrodes. NCV were calculated automatically based on the two stimulation sites.

### Clinical and behavioral animal testing

#### Experimental groups

At d80, which was considered as a definitive symptomatic stage based on the preceding electrophysiological analysis, mice were allocated to three treatment groups (Fas30, Fas100 and Veh) in an observer-blinded fashion. Groups were constituted to minimize inter-group variability by matching of animals with respect to body weight and litter.

#### Monitoring of disease

##### Neurological score

Neurological scores were assessed every three days for each mouse from 80 days of age. The neurological score employed a scale of 0–4 which had been developed in ALS mouse trials before (Weydt et al., 2003). *Score criteria* used to assign each score level were:

4: Full extension of hind legs away from lateral midline when mouse is suspended by its tail, and mouse can hold this for 2 s, suspended 2–3 times.

3: Collapse or partial collapse of leg extension toward lateral midline (weakness) or trembling of hind legs during tail suspension. Score criterion 3 is defined as clinical disease onset.

2: Toes curl under at least twice during walking of 10 cm, or any part of foot is dragging along cage bottom/table. From this score criterion on food pellets are left on bedding and water is additionally placed in a well on the bedding.

1: Rigid paralysis or minimal joint movement, foot not being used for forward motion.

0: Mouse cannot right itself within 30 s from either side or have lost 25% of their maximum body weight. This indicated fulfillment of the experimental termination criteria and mice were sacrificed.

##### Body weight

Body weight is a sensitive indicator of any malaise that might result from chronic drug treatment and of motor impairment that occurs during disease progression. Body weight measurements were recorded every 3 days for each animal beginning at 80 days of age.

##### Survival

Date and cause of death were recorded for each mouse. For ethical reasons, animals are closely monitored and sacrificed as moribund prior to actual death using criteria for severe illness. To determine duration of survival reliably and ethically, the moribund state, defined as the inability of mice to right themselves 30 s after being placed on a side or have lost 25% of their maximum body weight (a neurological score of 0) was used. The moribund mice were scored as “dead,” and were sacrificed.

##### Rotarod test

The rotarod apparatus (Ugo Basile, Comerio, Italy) was used to measure motor coordination, balance, and motor learning ability (Miana-Mena et al., 2005; Crawley, 2008). A good performance requires a high degree of sensorimotor coordination. It consists of a computer-controlled motor-driven rotating spindle and five lanes for five mice. Falls of the mice are detected automatically by pressure on a plastic plate at the bottom. After training for three times at a constant speed of 15 rpm and for the duration of 180 s, mice were tested every 3 days beginning on d80 and the time for which an animal can remain on the rotating rod is measured. For animals which could not remain 180 s on the rotarod, a second and third trial with a break of 10 min was applied. Means of all trials of each animal were included in the statistical analysis. The time of 180 s is chosen as an arbitrary cut-off time because the majority of significant differences in motor coordination are detected in this time frame.

### Immunohistochemistry and quantification of motoneurons and microglia

Animals were killed by CO_2_ insufflation at disease end-stage in accordance with the local guidelines. Thereafter, they were transcardially perfused with PBS solution followed by 4% paraformaldehyde. Spinal cords were removed as described before (Günther et al., 2012) and were post-fixed for 2 h in 4% paraformaldehyde. Thereafter, the tissue was dehydrated in 30% sucrose overnight and kept at −20°C until further processing. Coronal sections of the lumbar spinal cord (L3–L6) with a thickness of 20 μm were prepared using a cryostat (Leica, Wetzlar, Germany) and collected on gelatine-coated glass slides.

Spinal cord sections were immunolabeled with the primary antibodies (anti-ChAT, 1:100, Millipore; anti-Iba1, 1:500, WAKO, Osaka, Japan) and subsequently with the respective secondary antibodies (Cy3 anti-goat or Cy2/Dylight anti-rabbit; both Dianova).

In order to quantify MN numbers in the ventral horn of coronal cryosections from the lumbar spinal cord, cells in the ventral horn were counted as MN if they had a clearly identifiable nucleolus, were at least 200 μm^2^ in size and were ChAT-positive. At least ten sections per mouse spinal cord including both ventral horns being at least 100 μm apart over a length of at least 1000 μm from the lumbar spinal cord were counted. In the treatment groups a total number of nine mice were analyzed from vehicle (*n* = 3), Fas30 (*n* = 3) and Fas100 (*n* = 3). Microgliosis was evaluated by the quantification of Iba1 positive cells in the ventral horn of lumbar spinal cord sections. In contrast to the MN analysis MG were not confined to ventral horn region. Therefore, the ventral horn area was measured and Iba1 positive cells were manually counted and plotted as cell numbers per 0.2 mm^2^. A total number of nine mice each gender was analyzed from vehicle (*n* = 3), Fas30 (*n* = 3) and Fas100 (*n* = 3). Means of both ventral horns were calculated and the mean of all sections of each animal was included in the final statistical analysis for both ChAT and Iba1 cell numbers.

### Statistical analysis

For the analysis of electrophysiological quantifications between two groups the unpaired Student's *t*-test was applied. Multiple group comparisons of immunohistological cell counts were done by one-way ANOVA followed by Tukey's *post-hoc* test. Behavioral data were subjected to a multifactorial ANOVA analysis and *post-hoc* Fisher's test. Survival data were analyzed using Kaplan-Meier survival fit analysis with Log-Rank tests for statistical significance. Statistical analyses were performed using Statistica 10.0 (StatSoft, Hamburg, Germany) and Kyplot 2.0 (KyensLab Inc, Tokyo, Japan). Data are represented as mean ± standard error of the mean. Differences were considered significant with ^*^P < 0.05; ^**^P < 0.01; ^***^P < 0.001.

## Results

### Electrophysiological alterations indicate onset of disease

**Figure 1:**
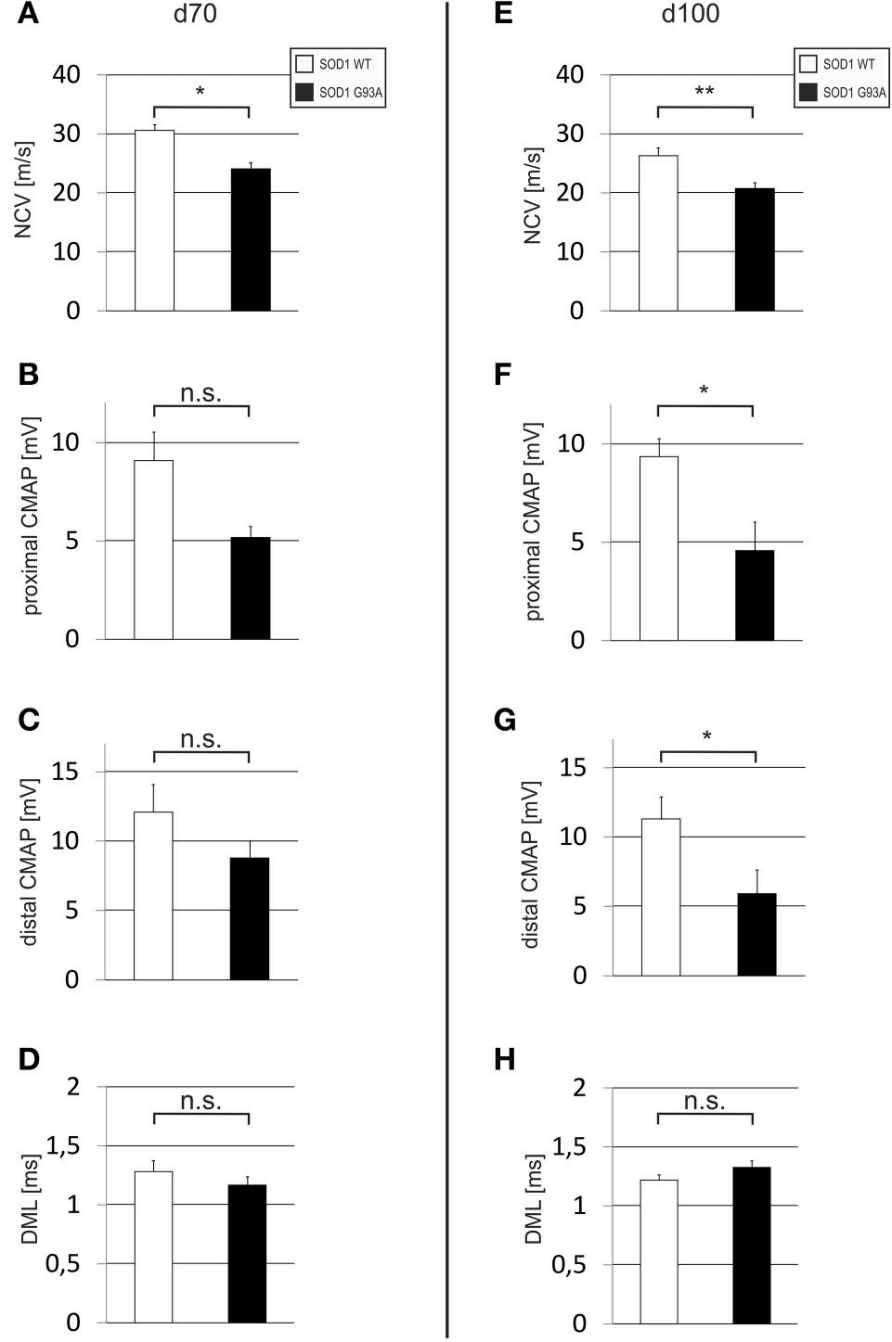
**Electrophysiological analysis including NCV, CMAP and DML of the sciatic nerve**. Depicted are histograms for d70 **(A–D)** and for d100 old **(E–H)** SOD1^WT^ and SOD1 ^G93A^ mice (SOD1^WT^ d70 *n* = 3, SOD1 ^G93A^ d70 *n* = 3, SOD1^WT^ d100 *n* = 6, SOD1 ^G93A^ d100 *n* = 6). Bar represents means ± SEM; ^*^P < 0.05.; ^**^P < 0.01.

In order to detect an early impairment of motor nerve function as indicator for disease onset we examined wildtype and SOD1^G93A^ mice with electrophysiological methods at day of life 70 (d70) and at day of life 100 (d100). At d70, the NCV of the sciatic nerve was already significantly delayed in SOD1^G93A^ animals if compared to wildtype controls (SOD1^WT^: 30.63 ± 0.95 m/s; SOD1^G93A^: 24.07 ± 1.10 m/s) (Figure 1A). This NCV difference was also persistent at d100 (SOD1^WT^: 26.31 ± 1.33 m/s; SOD1^G93A^: 20.72 ± 0.98 m/s) (Figure 1E). The CMAP at d70 was reduced in SOD1^G93A^ mice in comparison to SOD1^WT^ [proximal CMAP: SOD1^WT^ 9.10 ± 1.44 mV; SOD1^G93A^ 5.17 ± 0.57 mV (Figure 1B); distal CMAP: SOD1^WT^ 12.10 ± 1.98 mV; SOD1^G93A^ 8.78 ± 1.23 mV (Figure 1C)]. At d100, this difference increased even more [proximal CMAP: SOD1^WT^ 9.35 ± 0.93 mV; SOD1^G93A^ 4.58 ± 1.45 mV (Figure 1F); distal CMAP: SOD1^WT^ 11.30 ± 1.61 mV; SOD1^G93A^ 5.91 ± 1.73 mV (Figure 1G)]. The DML was not significantly different at both time points [d70: SOD1^WT^ 1.28 ± 0.09 ms; SOD1^G93A^ 1.17 ± 0.07 ms (Figure 1D); d100: SOD1^WT^ 1.22 ± 0.05 ms; SOD1^G93A^ 1.33 ± 0.06 ms (Figure 1H)].

### Weight dynamics are not altered by fasudil treatment in SOD1^G93A^ mice

**Figure 2:**
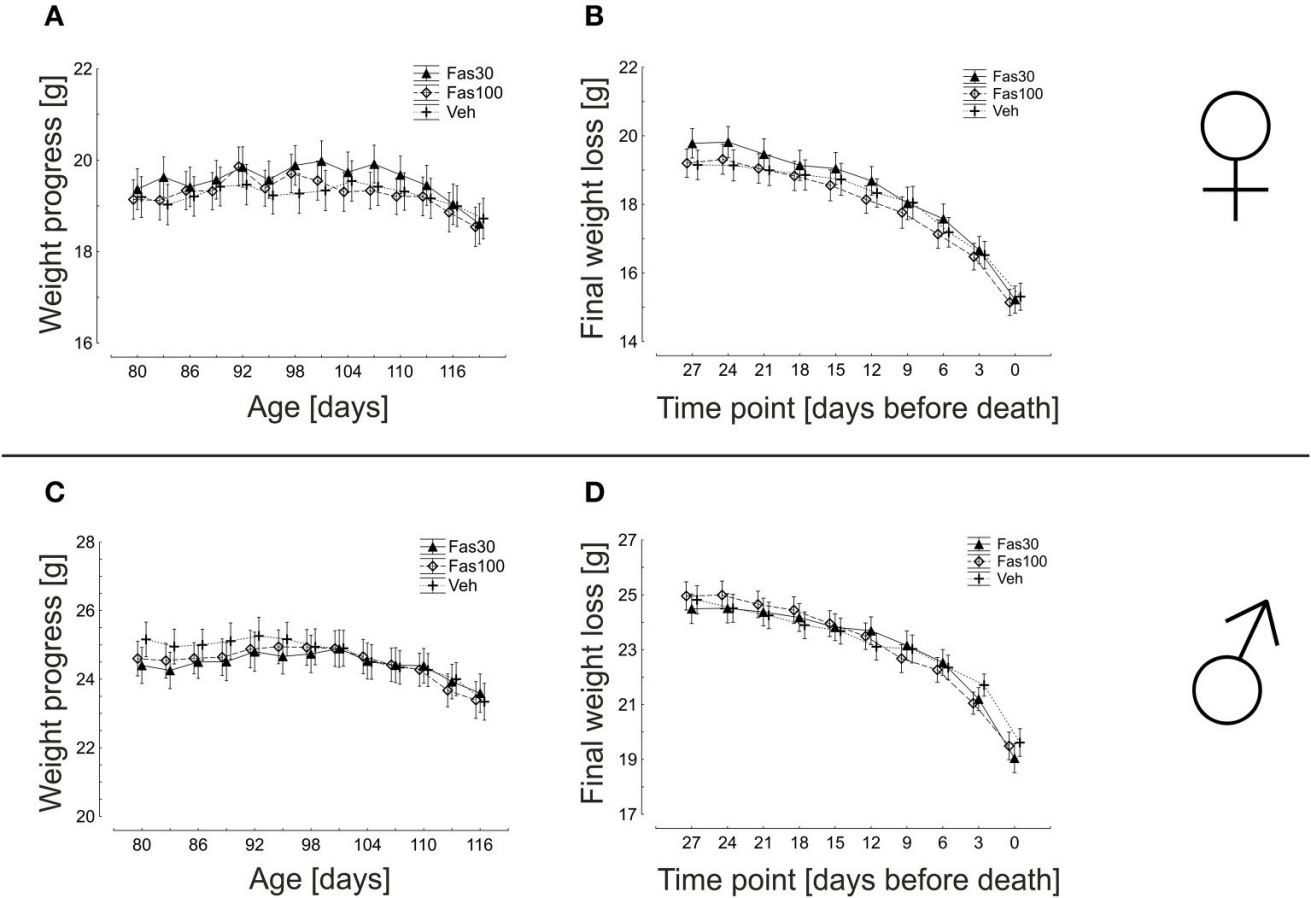
**Weight dynamics in SOD1^G93A^ transgenic mice orally treated with 30 mg/kg Fasudil (Fas30), 100 mg/kg Fasudil (Fas100) or with vehicle (Veh)**. Depicted are female (Fas30 *n* = 14, Fas100 *n* = 15, Veh *n* = 14) **(A,B)** and male (Fas30 *n* = 11, Fas100 *n* = 12, Veh *n* = 12) **(C,D**) cohorts.

Oral Fasudil treatment was initiated at d80 in symptomatic SOD1^G93A^ mice in two different dosages Fas30 and Fas100, both in female and in male mice. Veh-treated SOD1^G93A^ female and male cohorts served as control. Among all cohorts differentiated by gender, there were no significant differences in weight dynamics. In females, the weight maximum for the Veh group was reached at day 104 (19.54 ± 0.42 g), for the Fas30 group at day 101 (19.98 ± 0.42 g) and for the Fas100 group at day 92 (19.86 ± 0.46 g). This corresponds to a mean maximum weight gain from day 80 on in the Veh group of 1.94%, in the Fas30 group of 3.24% and in the Fas100 group of 3.90%. In males, the weight maximum for the Veh group was reached at day 92 (25.26 ± 0.48 g), for the Fas30 group at day 101 (24.89 ± 0.67 g) and for the Fas100 group at day 95 (24.94 ± 0.43 g) corresponding to a mean maximum weight gain from day 80 of 0.41% (Veh), 2.01% (Fas30) and 1.60% (Fas100). A progressive weight loss in the female veh group was observed starting on day 104 and in both female treatment groups starting on day 107. In contrast, progressive weight loss in males started on day 92 in veh group, on day 101 in the Fas30 group and on day 95 in the Fas100 group. The overall dynamics of weight loss did not significantly differ in female or male groups (Figure 2).

### Progression of neurological symptoms and overall survival are not modified by fasudil

**Figure 3:**
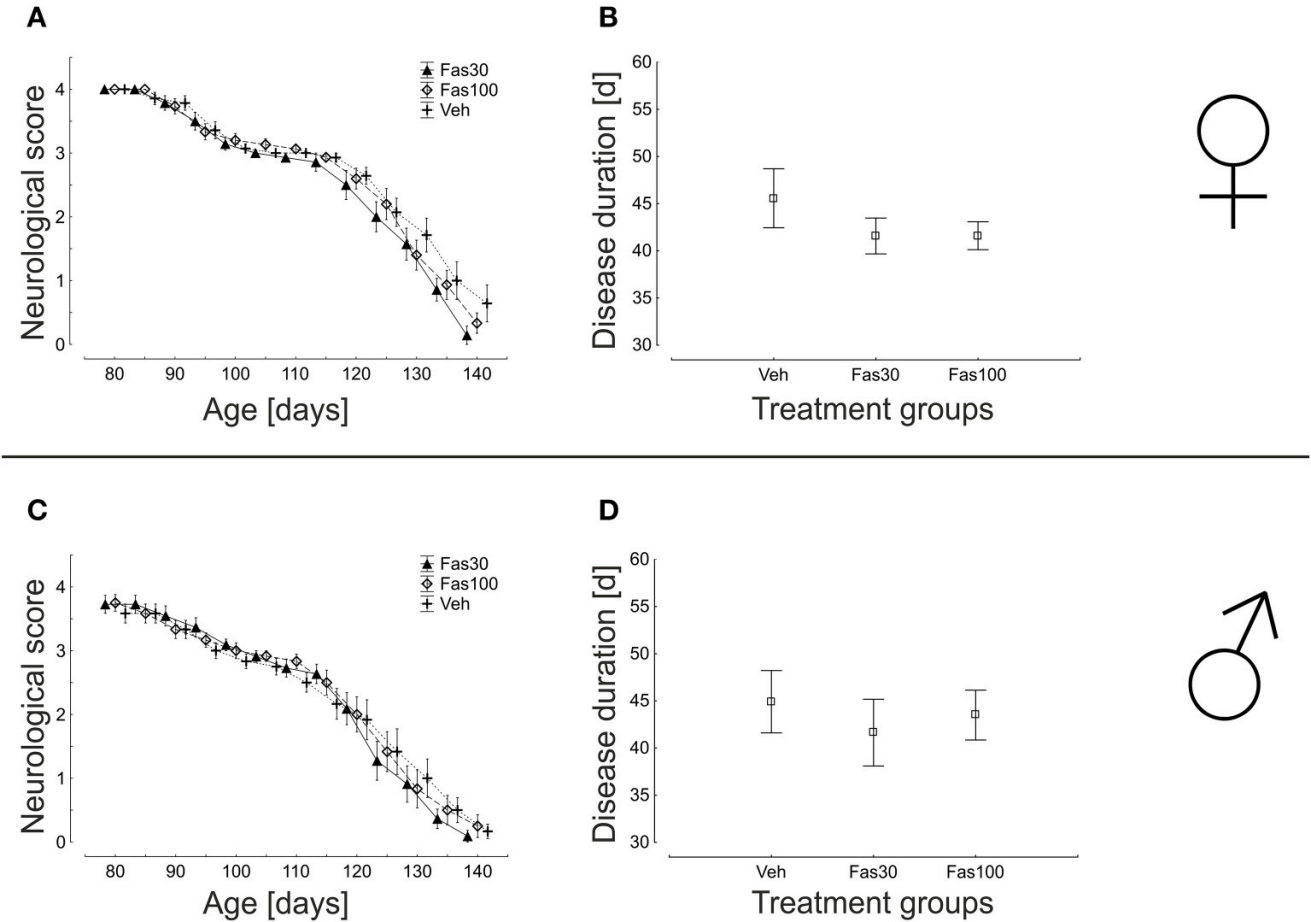
**Progression of neurological symptoms and disease duration in SOD1^G93A^ mice treated with 30 mg/kg Fasudil (Fas30), 100 mg/kg Fasudil (Fas100), or with vehicle (Veh) as control**. Displayed are neurological scores and disease duration for female (Fas30 *n* = 14, Fas100 *n* = 15, Veh *n* = 14) **(A,B)** and male (Fas30 *n* = 11, Fas100 *n* = 12, Veh *n* = 12) **(C,D)** cohorts.

We repeatedly performed neurological scoring over the entire treatment period. Here, application of Fas30 or Fas100 did not significantly modify the progression of clinical disease in both female and male mice (Figures 3A,C). Similarly, the average duration of disease was not significantly different between female (Veh 45,57 ± 3,12 d; Fas30 41,57 ± 1,89 d; Fas100 41,60 ± 1,48 d) and male (Veh 44,92 ± 3,31 d; Fas30 41,64 ± 3,54 d; Fas100 43,50 ± 2,64 d) treatment groups (Figures 3B,D).

**Figure 4:**
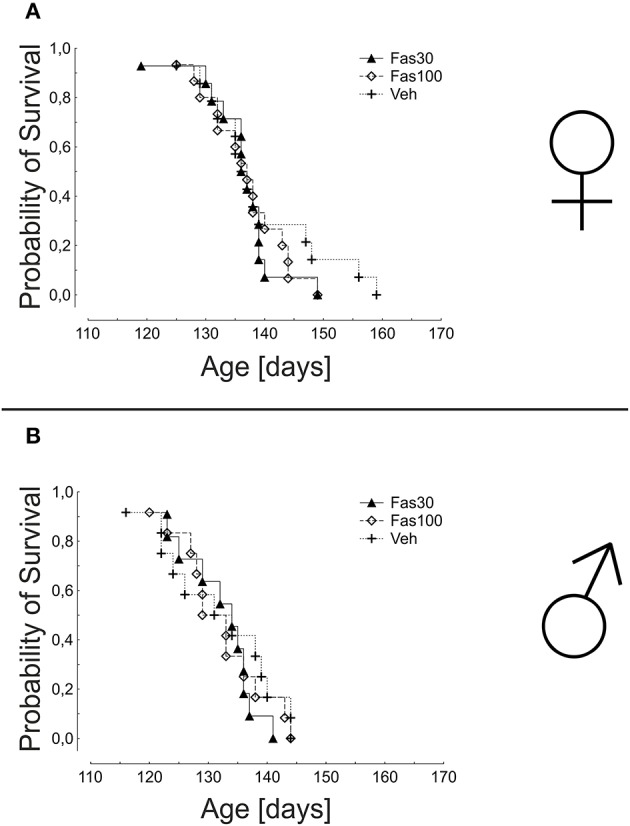
**Survival in SOD1^G93A^ mice treated with 30 mg/kg Fasudil (Fas30), 100 mg/kg Fasudil (Fas100), or with vehicle (Veh)**. Depicted is the cumulative probability of survival of female (Fas30 *n* = 14, Fas100 *n* = 15, Veh *n* = 14) **(A)** and male (Fas30 *n* = 11, Fas100 *n* = 12, Veh *n* = 12) **(B)** mice.

In the analysis of overall survival, the mean times of survival were not significantly different in any treatment groups, neither in female (Veh: 139.1 ± 2.7 days; Fas30 135.9 ± 1.8 days; Fas100 136.7 ± 1.7 days) nor in male mice (Veh: 131.7 ± 2.8 days; Fas30 131.9 ± 1.8 days; Fas100 131.9 ± 2.1 days) (Figure 4).

### Fasudil improves motor performance in male mice

**Figure 5:**
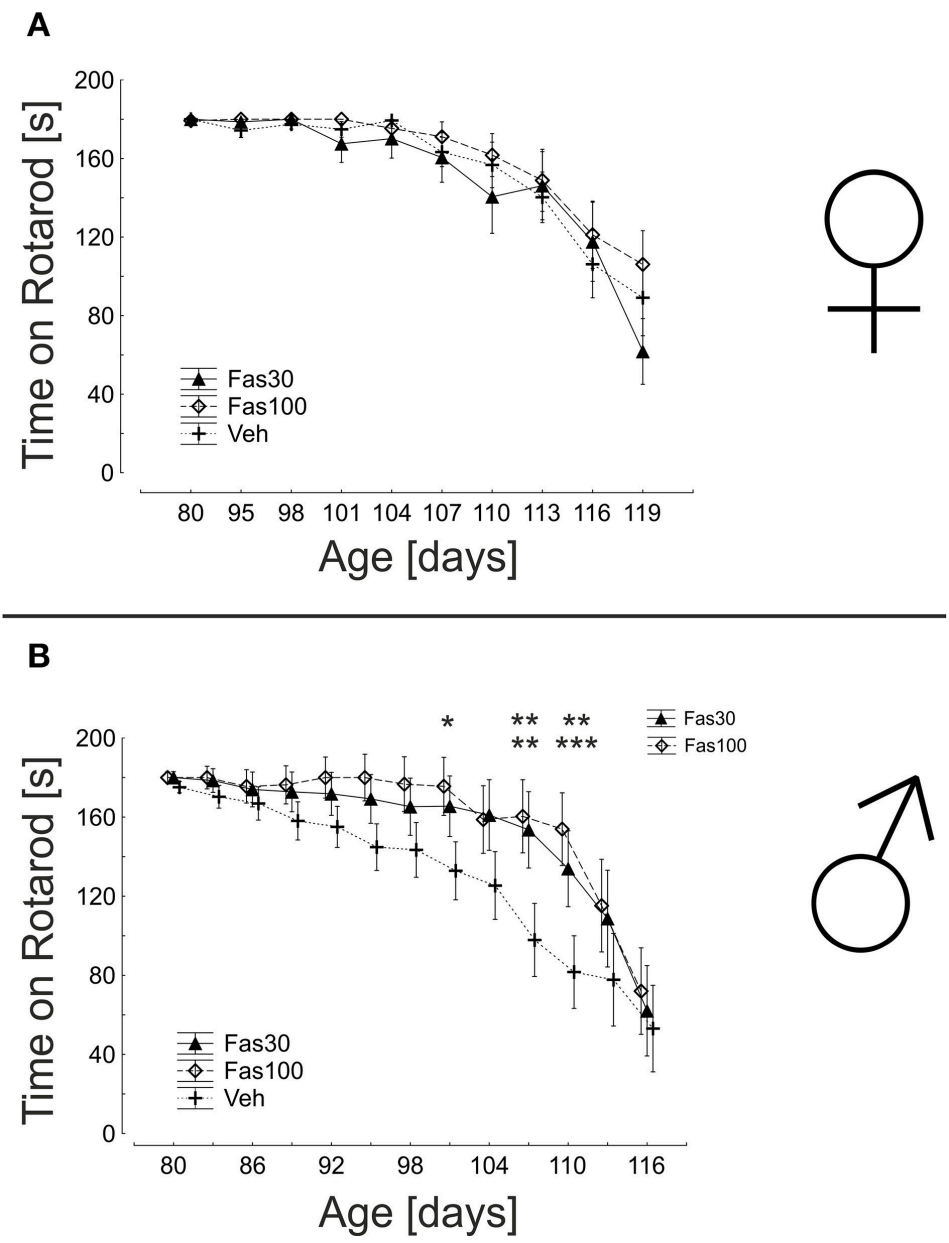
**ROCK inhibition with Fasudil stabilizes motor performance in SOD1^G93A^ male mice**. Displayed are mean durations on the rotarod of female (Fas30 *n* = 14, Fas100 *n* = 15, Veh *n* = 14) **(A)** and male (Fas30 *n* = 11, Fas100 *n* = 12, Veh *n* = 12). **(B)** SOD1 ^G93A^ mice treated with 30 mg/kg Fasudil (Fas30), 100 mg/kg Fasudil (Fas100), or with vehicle (Veh). Data represent means ± SEM. ^*^ *P* < 0.05; ^**^P < 0.01, ^***^P < 0.001.

The evaluation of motor performance on a rotarod device reflects both muscular strength and motor coordination. While female mice showed similar results in all treatment groups, male mice were significantly better in both Fas30 and Fas100 treatment groups. The most significant differences to the control group were observed at intermediate d110 (endurance on rotarod: Veh 81.64 ± 22.42 s; Fas30 133.91 ± 17.28 s; Fas100 153.92 ± 15.14 s) (Figure 5).

### Motoneuron cell survival and microglial infiltration at disease end-stage

**Figure 6:**
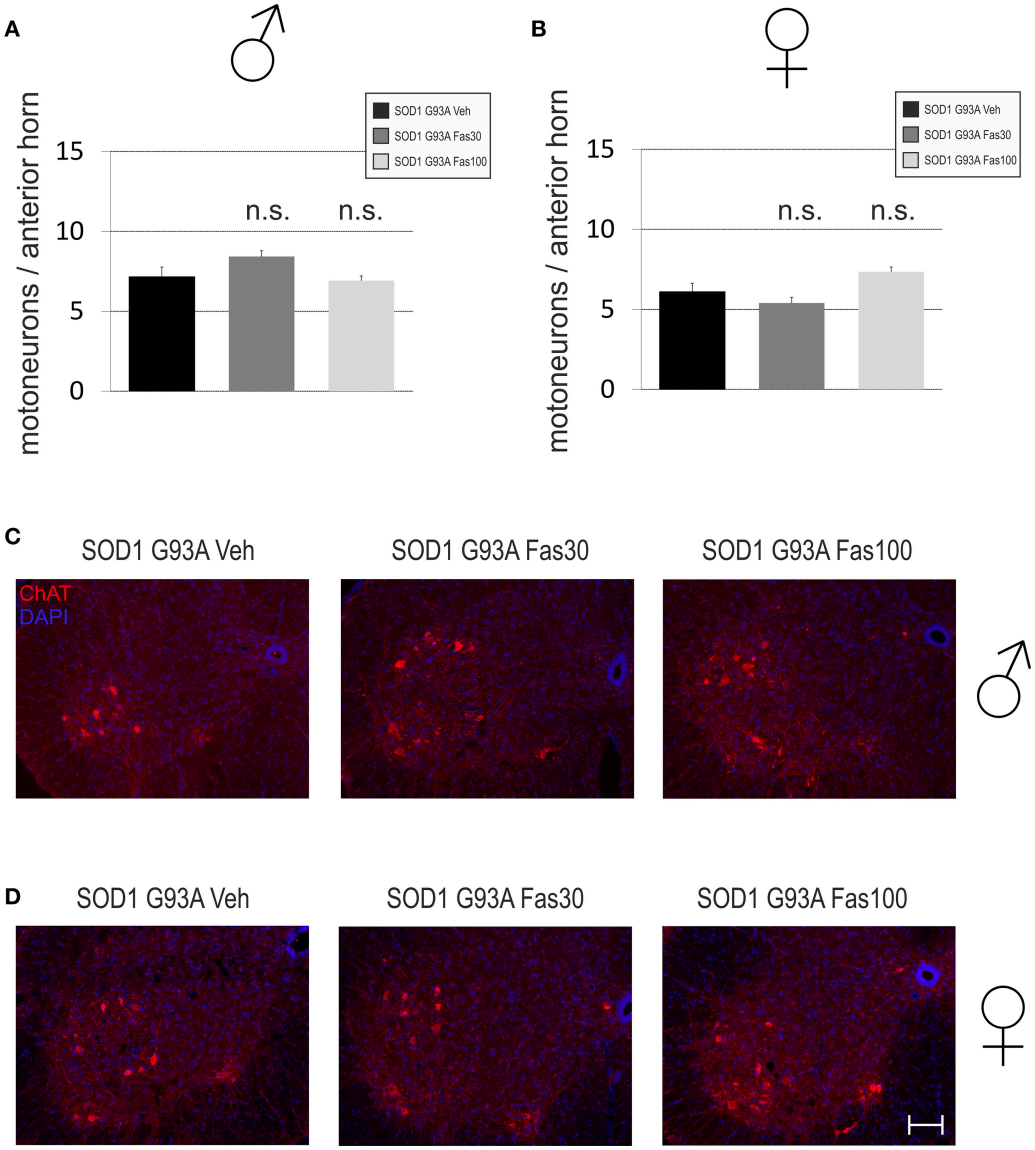
**Motoneuronal survival in the spinal cord in end-stage stage SOD1^G93A^ transgenic mice**. **(A,B)** Numbers of ChAT-immunopositive motoneurons (MN) per section in the spinal cord anterior horn of female (Fas30 *n* = 3, Fas100 *n* = 3, Veh *n* = 3) and male (Fas30 *n* = 3, Fas100 *n* = 3, Veh) mice treated with 30 mg/kg Fasudil (Fas30), 100 mg/kg Fasudil (Fas100), or with vehicle (Veh). **(C,D)** Representative micrographs of ChAT-immunopositive MNs in the spinal cord anterior horn of female **(C)** and male **(D)** mice. [ChAT/Cy3 (red), DAPI (blue)]. Bars represent means ± SEM. Scale bar: 100 μm.

In order to evaluate underlying neuropathological alterations, we quantified MN survival and MG cell infiltration in the anterior horn of the lumbar spinal cord. There were no significant differences in the number of ChAT-immunopositive MN in both female (Veh 6.1 ± 0.53 MN/anterior horn; Fas30 5.39 ± 0.36 MN/anterior horn; Fas100 7.35 ± 0.29 MN/anterior horn) and male mice (ChAT numbers Veh 7.15 ± 0.61 MN/anterior horn; Fas30 8.43 ± 0.37 MN/anterior horn; Fas100 6.92 ± 0.29 MN/anterior horn) (Figure 6).

**Figure 7:**
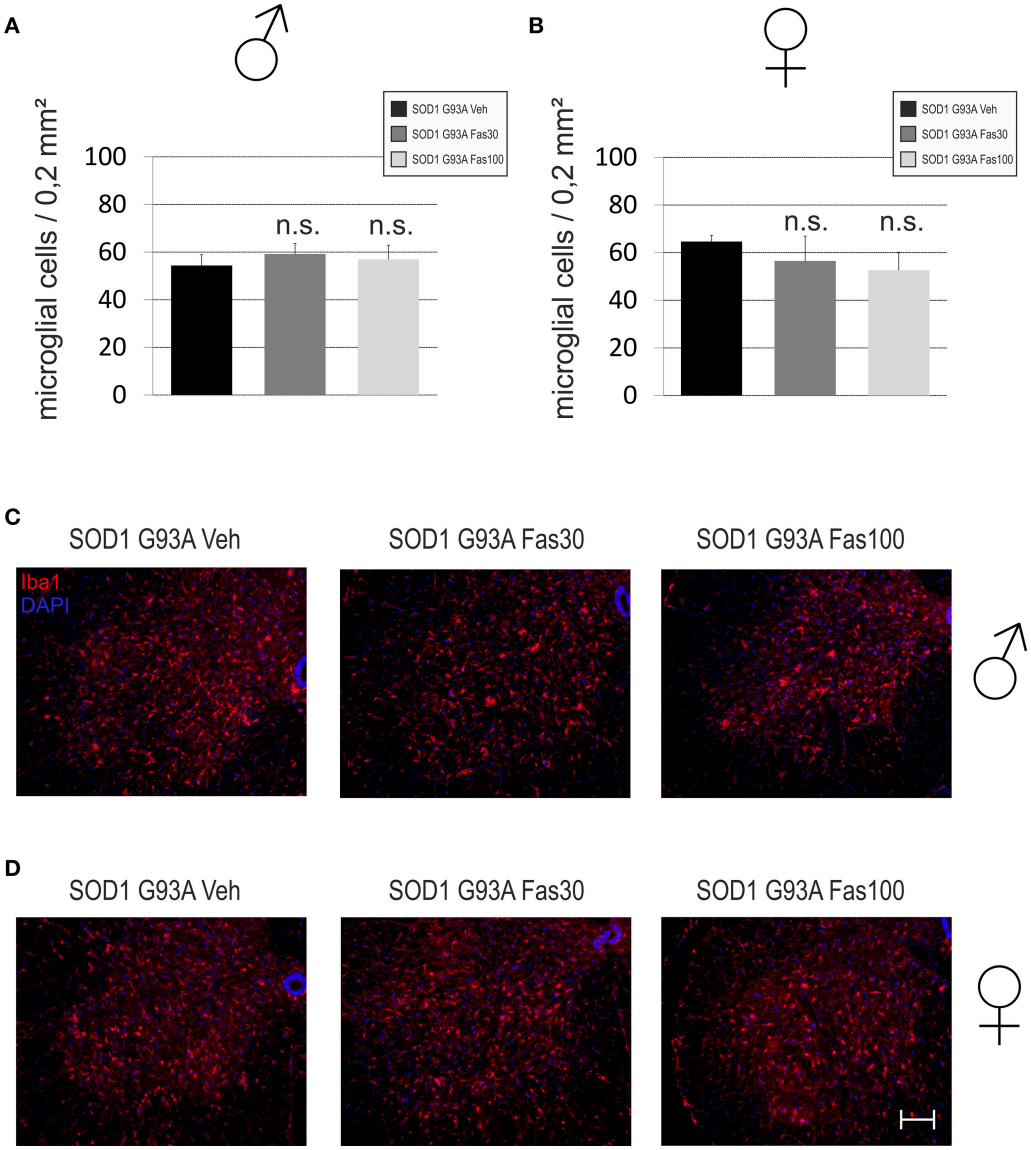
**Infiltration of microglial cells in the lumbar spinal cord anterior horn in end-stage stage SOD1^G93A^ mice**. **(A,B)** Numbers of Iba1-immunopositive microglia (MG) per section in the spinal cord anterior horn of female (Fas30 *n* = 3, Fas100 *n* = 3, Veh) **(A)** and male (Fas30 *n* = 3, Fas100 *n* = 3, Veh) **(B)** mice treated with 30 mg/kg Fasudil (Fas30), 100 mg/kg Fasudil (Fas100), or with vehicle (Veh). **(C,D)** Representative micrographs of Iba1-immunopositive MG in spinal cord anterior horn of female **(C)** and male **(D)** mice. [Iba1/Cy3 (red), DAPI (blue)]. Bars represent means ± SEM. Scale bar: 100 μm.

Iba1-immunopositive MG cells were found in the spinal cord anterior horn at similar numbers in all treatment groups in female (Veh 64.64 ± 2.65 MG/section; Fas30 56.52 ± 10.42 MG/section; Fas100 52.61 ± 7.53 MG/section) and male mice (Veh 54.38 ± 4.54 MG/section; Fas30 59.22 ± 4.41 MG/section; Fas100 56.91 ± 5.96 MG/section) (Figure 7).

## Discussion

ROCK inhibition by Fasudil has previously been shown to exert neuroprotective, but also pro-regenerative and axon-stabilizing effects in models of both ALS and PD (Tönges et al., 2012, 2014). In addition, improvement of neurological function or an increase of survival by ROCK inhibition was recently reported in animal models of Huntington's disease and spinal muscular atrophy (Li et al., 2009; Bowerman et al., 2010, 2012). Treatment of presymptomatic SOD1^G93A^ transgenic mice with Fasudil starting at day of life 50 increased survival and improved motor behavior in two independent studies (Takata et al., 2013; Tönges et al., 2014). However, no treatment with Fasudil has been performed so far in any of these models in an advanced disease stage. Therefore, we performed a treatment study in symptomatic SOD1^G93A^ mice in order to evaluate its therapeutic potential under clinically more relevant conditions.

### Disease onset in the SOD1^G93A^ mouse model

Several parameters can be used to indicate disease onset in mouse models of ALS. One parameter is the clinical phenotype of the mice. It is well described that around d80 first clinical symptoms start to appear with trembling of hind limbs (Turner and Talbot, 2008). Consequently, detection of trembling of hind limbs is included in a commonly used neurological scoring system for this mouse model (Miana-Mena et al., 2005) representing the first disease stage (Weydt et al., 2003). In previous studies, we had observed a decline from the healthy phenotype (score 4) to the first disease stage (score 3) between d80 and d100 in both male and female animals. At d100 nearly all animals had reached disease onset (score 3) (Günther et al., 2014). Furthermore, motor testing with a rotarod device resulted in a decline of performance between d80 and d100. Applying a hanging wire test we observed a decrease of muscle strength already after d70 (Günther et al., 2014; Tönges et al., 2014). Concerning these functional parameters it is important to note that male mice are impaired earlier than females with regard to clinical scoring and motor behavior. Taking into account the genetic background of the animals, differences between females and males in the SOD1^G93A^ have been previously observed (Oliván et al., 2015; Pfohl et al., 2015). While both, the C57BL/6 and the B6SJL background, lead to delayed disease onset in females, a prolonged survival was only observed in females with the B6SJL background in comparison to their male counterparts (Pfohl et al., 2015). Similar to previous reports, female animals in our study had a prolonged survival time and a delayed decline in motor performance as compared to their male counterparts (Miana-Mena et al., 2005; Alves et al., 2011; Mancuso et al., 2012; Pfohl et al., 2015). Another quantitative method to assess initiation or progression of disease is the examination based on electrophysiological measurements, which was used in this study and represents also an important diagnostic tool for human ALS (Brooks et al., 2000). Considerable differences between SOD1^WT^ and SOD1^G93A^ have recently been reported in a previous study on time point d100 and additional comprehensive evaluations of electrophysiological measurements have been done (Alves et al., 2011; Mancuso et al., 2012; Tönges et al., 2014). Mancuso et al. determined onset of disease between 70 and 90 days of age in the SOD1 mouse model which was associated with CMAP alterations of the tibialis anterior muscle suggesting that electrophysiological measures can be a marker for disease onset (Mancuso et al., 2012). In our own electrophysiological analysis we also used neurographic measurements and could detect deteriorations in CMAPs and a significant deceleration of NCVs compared to SOD1^WT^ already on d70. In line with progressive disease, CMAPs deteriorated and became significantly reduced at d100. These results confirm a disease onset in electrophysiological terms at around d70 and the presence of progressive disease until d100 in SOD1^G93A^ animals. In order to define a distinct starting point for treatment in our study we then chose d80 which additionally incorporates interindividual variances in SOD1^G93A^ mice and thus represent a clear time point of manifest disease.

### Fasudil improves motor performance in male SOD1^G93A^ mice

Fasudil treatment was thus started at d80 in two different concentrations (Fas30 and Fas100) and was clinically well tolerated. The weight course was comparable to the Veh group. In our treatment paradigm, Fasudil treatment did not prolong survival and we could not observe a statistically relevant benefit in the graded clinical examination. However, if we applied more sensitive examination methods for motor strength and coordination with rotarod testing, male animals showed a benefit from treatment with both dosages.

Others and our own group had found a moderate protection of spinal cord MN in an intermediate analysis on day 100 with identical dosages of Fasudil if treatment was started already at a presymptomatic stage at day 50 (Takata et al., 2013; Tönges et al., 2014). However, the histological analysis in endstage animals did not reveal significant differences of MN survival or in MG infiltration between the groups. In the symptomatic treatment study from day 80, we similarly did not detect clear ameliorations such as an increased motoneuron survival on the neuropathological level. This may be due to an only functional improvement of motoneurons, which, however, was too small or unstable to transfer to a persistant cellular change.

Another reason for the rather small treatment effect observed in our study is most probably the advanced pathology that is already present at the time, when animals become clinically symptomatic (Turner and Talbot, 2008). It is also known, that progression of disease in the SOD1^G93A^ mouse model is rather rapid and one could hypothesize that a more slowly progressing disease model would yield more favorable results. Many groups have performed therapeutic pilot studies in the SOD1^G93A^ mouse model in a clinically presymptomatic stage between 30 and 60 days of age, long before clinical symptoms become obvious (Turner and Talbot, 2008), but this strategy is insufficient if no verification with a treatment after symptom onset is performed.

Of interest, significant, but overall only moderate therapeutic effects were found exclusively in male mice in this study. Gender specific effects have also been reported in the Y-27632 presymptomatic treatment study (Günther et al., 2014) and differences in the disease course between male and female mice were previously described (Alves et al., 2011; Oliván et al., 2015; Pfohl et al., 2015). Ovariectomy in the SOD1^G93A^ mouse model leads to a decrease in survival time and makes female mice progress almost as fast as male mice. In line with this finding, estrogen treatment can counteract the effects of ovariectomy (Groeneveld et al., 2004; Choi et al., 2008). Similarly, ovariectomy in the MPTP model of Parkinson's disease leads to an increase of ROCK activity and treatment with either estrogen or a ROCK inhibitor could inhibit MPTP-induced dopaminergic cell death. Furthermore, ROCK activity was less increased in ovariectomized MPTP mice when treated with estrogen (Rodriguez-Perez et al., 2013). In analogy, ROCK activity could be higher in male mice in advanced disease and therefore the application of Fasudil could have been more successful in male mice in our study.

In multiple other neurodegenerative disease models, ROCK inhibitors and particularly Fasudil showed very promising results. In the MPTP model of Parkinson's disease Fasudil significantly protected both dopaminergic neurons and its axonal nigrostriatal terminals, restoring striatal neurotransmitter levels and attenuating behavioral deficits (Tönges et al., 2012). Furthermore, Fasudil attenuated alpha-synuclein aggregation *in vitro* and *in vivo* in a genetic model of Parkinson's disease (Tatenhorst et al., 2016). In a Drosophila model of tauopathy, Fasudil suppressed the rough eye phenotype and mitigated pathogenic tau levels by inducing autophagic pathways (Gentry et al., 2016). Furthermore, significant therapeutic effects were also observed in a mouse model of Huntington's disease, both with Y-27632 and Fasudil treatment (Li et al., 2009, 2013), and in a model of spinal muscle atrophy, a condition with high similarity to ALS. Here the clinical progression was attenuated by Y-27632 and Fasudil (Bowerman et al., 2010, 2012).

In a translational perspective, Fasudil combines several highly promising features. It is an already licensed drug, which has a long-standing clinical history with several tens of thousands of patients treated, demonstrating a favorable safety-profile (Suzuki et al., 2007). Its pharmacokinetics is characterized by excellent solubility in water, sufficient uptake into the CNS and no major interactions with Cytochrome P450 enzymes. The fact that Fasudil is effective in several disease models, which all combine similar pathomechanisms of neurodegeneration, reaffirms its potential for a drug-repositioning approach.

## Conclusion

The therapeutic application of the ROCK inhibitor Fasudil in an advanced symptomatic disease stage in SOD1^G93A^ ALS mice resulted in significant improvements of motor function, but was less effective when compared to paradigms, when animals were treated before clinical symptom onset. However, given the advanced pathology at first symptom onset in this model, the ability to beneficially modify motor function is remarkable. Although the neuroprotective potential of Fasudil in ALS is best if applied before clinical symptom onset, our study supports the testing of Fasudil in clinical trials including patients at the earliest-possible disease stage.

## Author contributions

RG: Conception and design, collection and assembly of data, data analysis, and interpretation, manuscript drafting. AB, JK, TN, MS, and MB: Collection and/or assembly of data, critical revision of manuscript. LT and PL: Conception and design, principal investigator, collection, and assembly of data, data analysis and interpretation, manuscript drafting.

### Conflict of interest statement

LT and PL have filed a priority patent application on the use of Fasudil for the treatment of ALS. The other authors declare that the research was conducted in the absence of any commercial or financial relationships that could be construed as a potential conflict of interest.

## Abbreviations

ANOVA: analysis of variance
ALS: amyotrophic lateral sclerosis
CMAP: compound muscle action potential
CNS: central nervous system
d70: day of life 70
d80: day of life 80
d100: day of life 100
DML: distal motor latency
Fas30: Fasudil 30 mg/kg body weight
Fas100: Fasudil 100 mg/kg body weight
FUS: Fused in sarcoma
MN: motoneuron
MG: microglial cells
NCV: Nerve conduction velocity
PBS: phosphate-buffered solution
ROCK: rho-associated kinase
SMA: spinal muscular atrophy
SOD1: superoxide dismutase 1
SOD1^G93A^: superoxide dismutase 1 G93A transgenic
SOD1^WT^: superoxide dismutase 1 wild type
TDP-43: TAR DNA-binding protein 43
Veh: vehicle (drinking water without supplementation).

## References

[B1] Alves C. J. de Santana L. P. dos Santos A. J. de Oliveira G. P. Duobles T. Scorisa J. M.. (2011). Early motor and electrophysiological changes in transgenic mouse model of amyotrophic lateral sclerosis and gender differences on clinical outcome. Brain Res. 1394, 90–104. 10.1016/j.brainres.2011.02.060 21354109

[B2] Benatar M. (2007). Lost in translation: treatment trials in the SOD1 mouse and in human ALS. Neurobiol. Dis. 26, 1–13. 10.1016/j.nbd.2006.12.015 17300945

[B3] Bermel C. Tonges L. Planchamp V. Gillardon F. Weishaupt J. H. Dietz G. P.. (2009). Combined inhibition of Cdk5 and ROCK additively increase cell survival, but not the regenerative response in regenerating retinal ganglion cells. Mol. Cell. Neurosci. 42, 427–437. 10.1016/j.mcn.2009.09.005 19782753

[B4] Bowerman M. Beauvais A. Anderson C. L. Kothary R. (2010). Rho-kinase inactivation prolongs survival of an intermediate SMA mouse model. Hum. Mol. Genet. 19, 1468–1478. 10.1093/hmg/ddq021 20097679

[B5] Bowerman M. Murray L. M. Boyer J. G. Anderson C. L. Kothary R. (2012). Fasudil improves survival and promotes skeletal muscle development in a mouse model of spinal muscular atrophy. BMC Med. 10:24. 10.1186/1741-7015-10-24 22397316 PMC3310724

[B6] Brites D. Vaz A. R. (2014). Microglia centered pathogenesis in ALS: insights in cell interconnectivity. Front. Cell. Neurosci.8:117. 10.3389/fncel.2014.00117 24904276 PMC4033073

[B7] Brooks B. R. Miller R. G. Swash M. Munsat T. L. (2000). El Escorial revisited: revised criteria for the diagnosis of amyotrophic lateral sclerosis. Amyotroph. Lateral Sclerosis Other Motor Neuron Disord. 1, 293–299. 11464847 10.1080/146608200300079536

[B8] Choi C. I. Lee Y. D. Gwag B. J. Cho S. I. Kim S. S. Suh-Kim H. (2008). Effects of estrogen on lifespan and motor functions in female hSOD1 G93A transgenic mice. J. Neurol. Sci. 268, 40–47. 10.1016/j.jns.2007.10.024 18054961

[B9] Conti A. Riva N. Pesca M. Iannaccone S. Cannistraci C. V. Corbo M.. (2014). Increased expression of Myosin binding protein H in the skeletal muscle of amyotrophic lateral sclerosis patients. Biochim. Biophys. Acta 1842, 99–106. 10.1016/j.bbadis.2013.10.013 24184715

[B10] Crawley J. N. (2008). Behavioral phenotyping strategies for mutant mice. Neuron 57, 809–818. 10.1016/j.neuron.2008.03.001 18367082

[B11] Gentry E. G. Henderson B. W. Arrant A. E. Gearing M. Feng Y. Riddle N. C.. (2016). Rho kinase inhibition as a therapeutic for progressive supranuclear palsy and corticobasal degeneration. J. Neurosci. 36, 1316–1323. 10.1523/JNEUROSCI.2336-15.2016 26818518 PMC4728727

[B12] Golko-Perez S. Amit T. Youdim M. B. Weinreb O. (2016). Beneficial effects of multitarget iron chelator on central nervous system and gastrocnemius muscle in SOD1(G93A) transgenic ALS mice. J. Mol. Neurosci. 59, 504–510. 10.1007/s12031-016-0763-2 27173029

[B13] Groeneveld G. J. Van Muiswinkel F. L. Sturkenboom J. M. Wokke J. H. Bär P. R. Van den Berg L. H. (2004). Ovariectomy and 17beta-estradiol modulate disease progression of a mouse model of ALS. Brain Res. 1021, 128–131. 10.1016/j.brainres.2004.06.024 15328040

[B14] Günther R. (2015). Pharmacological Inhibition of Rho-Kinase in the Mouse Model of Amyotrophic Lateral Sclerosis. Dissertation, University of Göttingen, Göttingen.

[B15] Günther R. Saal K. A. Suhr M. Scheer D. Koch J. C. Bahr M.. (2014). The rho kinase inhibitor Y-27632 improves motor performance in male SOD1(G93A) mice. Front. Neurosci. 8:304. 10.3389/fnins.2014.00304 25339858 PMC4187656

[B16] Günther R. Suhr M. Koch J. C. Bahr M. Lingor P. Tonges L. (2012). Clinical testing and spinal cord removal in a mouse model for amyotrophic lateral sclerosis (ALS). J. Vis. Exp. 61:e3936. 10.3791/3936 22453893 PMC3415170

[B17] Gurney M. E. Pu H. Chiu A. Y. Dal Canto M. C. Polchow C. Y. Alexander D. D.. (1994). Motor neuron degeneration in mice that express a human Cu, Zn superoxide dismutase mutation. Science 264, 1772–1775. 10.1126/science.8209258 8209258

[B18] Hattori T. Shimokawa H. Higashi M. Hiroki J. Mukai Y. Tsutsui H.. (2004). Long-term inhibition of Rho-kinase suppresses left ventricular remodeling after myocardial infarction in mice. Circulation 109, 2234–2239. 10.1161/01.CIR.0000127939.16111.58 15096457

[B19] Jacobs M. Hayakawa K. Swenson L. Bellon S. Fleming M. Taslimi P.. (2006). The structure of dimeric ROCK I reveals the mechanism for ligand selectivity. J. Biol. Chem. 281, 260–268. 10.1074/jbc.M508847200 16249185

[B20] Léger B. Vergani L. Sorarù G. Hespel P. Derave W. Gobelet C.. (2006). Human skeletal muscle atrophy in amyotrophic lateral sclerosis reveals a reduction in Akt and an increase in atrogin-1. FASEB J. 20, 583–585. 10.1096/fj.05-5249fje 16507768

[B21] Li M. Huang Y. Ma A. A. Lin E. Diamond M. I. (2009). Y-27632 improves rotarod performance and reduces huntingtin levels in R6/2 mice. Neurobiol. Dis. 36, 413–420. 10.1016/j.nbd.2009.06.011 19591939

[B22] Li M. Yasumura D. Ma A. A. Matthes M. T. Yang H. Nielson G.. (2013). Intravitreal administration of HA-1077, a ROCK inhibitor, improves retinal function in a mouse model of huntington disease. PLoS ONE 8:e56026. 10.1371/journal.pone.0056026 23409115 PMC3569418

[B23] Lingor P. Tönges L. Pieper N. Bermel C. Barski E. Planchamp V.. (2008). ROCK inhibition and CNTF interact on intrinsic signalling pathways and differentially regulate survival and regeneration in retinal ganglion cells. Brain 131, 250–263. 10.1093/brain/awm284 18063589

[B24] Mancuso R. Oliván S. Mancera P. Pastén-Zamorano A. Manzano R. Casas C.. (2012). Effect of genetic background on onset and disease progression in the SOD1-G93A model of amyotrophic lateral sclerosis. Amyotroph. Lateral Sclerosis 13, 302–310. 10.3109/17482968.2012.662688 22424126

[B25] Miana-Mena F. J. Muñoz M. J. Yagüe G. Mendez M. Moreno M. Ciriza J.. (2005). Optimal methods to characterize the G93A mouse model of ALS. Amyotroph. Lateral Sclerosis Other Motor Neuron Disord. 6, 55–62. 10.1080/14660820510026162 16036427

[B26] Moloney E. B. de Winter F. Verhaagen J. (2014). ALS as a distal axonopathy: molecular mechanisms affecting neuromuscular junction stability in the presymptomatic stages of the disease. Front. Neurosci. 8:252. 10.3389/fnins.2014.00252 25177267 PMC4132373

[B27] Oliván S. Calvo A. C. Rando A. Muñoz M. J. Zaragoza P. Osta R. (2015). Comparative study of behavioural tests in the SOD1G93A mouse model of amyotrophic lateral sclerosis. Exp. Anim. 64, 147–153. 10.1538/expanim.14-0077 25736480 PMC4427729

[B28] Peters O. M. Ghasemi M. Brown R. H. Jr. (2015). Emerging mechanisms of molecular pathology in ALS. J. Clin. Invest. 125, 1767–1779. 10.1172/JCI71601 25932674 PMC4463186

[B29] Pfohl S. R. Halicek M. T. Mitchell C. S. (2015). Characterization of the contribution of genetic background and gender to disease progression in the SOD1 G93A mouse model of amyotrophic lateral sclerosis: a meta-analysis. J. Neuromuscul. Dis. 2, 137–150. 10.3233/JND-140068 26594635 PMC4652798

[B30] Philips T. Robberecht W. (2011). Neuroinflammation in amyotrophic lateral sclerosis: role of glial activation in motor neuron disease. Lancet Neurol. 10, 253–263. 10.1016/S1474-4422(11)70015-1 21349440

[B31] Planchamp V. Bermel C. Tönges L. Ostendorf T. Kügler S. Reed J. C.. (2008). BAG1 promotes axonal outgrowth and regeneration *in vivo* via Raf-1 and reduction of ROCK activity. Brain 131, 2606–2619. 10.1093/brain/awn196 18757464

[B32] Robberecht W. Philips T. (2013). The changing scene of amyotrophic lateral sclerosis. Nat. Rev. Neurosci. 14, 248–264. 10.1038/nrn3430 23463272

[B33] Rodriguez-Perez A. I. Dominguez-Meijide A. Lanciego J. L. Guerra M. J. Labandeira-Garcia J. L. (2013). Inhibition of Rho kinase mediates the neuroprotective effects of estrogen in the MPTP model of Parkinson's disease. Neurobiol. Dis. 58, 209–219. 10.1016/j.nbd.2013.06.004 23774254

[B34] Rosen D. R. Siddique T. Patterson D. Figlewicz D. A. Sapp P. Hentati A.. (1993). Mutations in Cu/Zn superoxide dismutase gene are associated with familial amyotrophic lateral sclerosis. Nature 362, 59–62. 10.1038/362059a0 8446170

[B35] Stewart A. Sandercock J. Bryan S. Hyde C. Barton P. M. Fry-Smith A.. (2001). The clinical effectiveness and cost-effectiveness of riluzole for motor neurone disease: a rapid and systematic review. Health Technol. Assess. 5, 1–97. 10.3310/hta5020 11809124

[B36] Suzuki Y. Shibuya M. Satoh S. Sugimoto Y. Takakura K. (2007). A postmarketing surveillance study of fasudil treatment after aneurysmal subarachnoid hemorrhage. Surg. Neurol. 68, 126–131; discussion: 31–32. 10.1016/j.surneu.2006.10.037 17586012

[B37] Takata M. Tanaka H. Kimura M. Nagahara Y. Tanaka K. Kawasaki K.. (2013). Fasudil, a rho kinase inhibitor, limits motor neuron loss in experimental amyotrophic lateral sclerosis models. Br. J. Pharmacol. 170, 341–351. 10.1111/bph.12277 23763343 PMC3834758

[B38] Tatenhorst L. Eckermann K. Dambeck V. Fonseca-Ornelas L. Walle H. Lopes da Fonseca T.. (2016). Fasudil attenuates aggregation of alpha-synuclein in models of Parkinson's disease. Acta Neuropathol. Commun. 4:39. 10.1186/s40478-016-0310-y 27101974 PMC4840958

[B39] Tönges L. Frank T. Tatenhorst L. Saal K. A. Koch J. C. Szego E. M.. (2012). Inhibition of rho kinase enhances survival of dopaminergic neurons and attenuates axonal loss in a mouse model of Parkinson's disease. Brain 135, 3355–3370. 10.1093/brain/aws254 23087045 PMC3501973

[B40] Tönges L. Gunther R. Suhr M. Jansen J. Balck A. Saal K. A.. (2014). Rho kinase inhibition modulates microglia activation and improves survival in a model of amyotrophic lateral sclerosis. Glia 62, 217–232. 10.1002/glia.22601 24311453

[B41] Tönges L. Koch J. C. Bahr M. Lingor P. (2011). ROCKing Regeneration: rho kinase inhibition as molecular target for neurorestoration. Front. Mol. Neurosci. 4:39. 10.3389/fnmol.2011.00039 22065949 PMC3207219

[B42] Turner B. J. Talbot K. (2008). Transgenics, toxicity and therapeutics in rodent models of mutant SOD1-mediated familial ALS. Prog. Neurobiol. 85, 94–134. 10.1016/j.pneurobio.2008.01.001 18282652

[B43] Turner M. R. Hardiman O. Benatar M. Brooks B. R. Chio A. de Carvalho M.. (2013). Controversies and priorities in amyotrophic lateral sclerosis. Lancet Neurol. 12, 310–322. 10.1016/S1474-4422(13)70036-X 23415570 PMC4565161

[B44] Vucic S. Rothstein J. D. Kiernan M. C. (2014). Advances in treating amyotrophic lateral sclerosis: insights from pathophysiological studies. Trends Neurosci. 37, 433–442. 10.1016/j.tins.2014.05.006 24927875

[B45] Weydt P. Hong S. Y. Kliot M. Moller T. (2003). Assessing disease onset and progression in the SOD1 mouse model of ALS. Neuroreport 14, 1051–1054. 10.1097/00001756-200305230-00029 12802201

[B46] Zhao Y. F. Zhang Q. Xi J. Y. Li Y. H. Ma C. G. Xiao B. G. (2015). Multitarget intervention of Fasudil in the neuroprotection of dopaminergic neurons in MPTP-mouse model of Parkinson's disease. J. Neurol. Sci. 353, 28–37. 10.1016/j.jns.2015.03.022 25908255

[B47] Zhao Y. Zhang Q. Xi J. Xiao B. Li Y. Ma C. (2015). Neuroprotective effect of fasudil on inflammation through PI3K/Akt and Wnt/beta-catenin dependent pathways in a mice model of Parkinson's disease. Int. J. Clin. Exp. Pathol. 8, 2354–2364. Available online at: www.ijcep.com/ISSN:1936-2625/IJCEP0005365 26045742 PMC4440051

[B48] Zielasek J. Martini R. Toyka K. V. (1996). Functional abnormalities in P0-deficient mice resemble human hereditary neuropathies linked to P0 gene mutations. Muscle Nerve 19, 946–952. 10.1002/(SICI)1097-4598(199608)19:8<946::AID-MUS2>3.0.CO;2-8 8756159

